# Multicenter Repeatability and Reproducibility of MR Fingerprinting in Phantoms and in Prostatic Tissue

**DOI:** 10.1002/mrm.29264

**Published:** 2022-06-17

**Authors:** Wei-Ching Lo, Leonardo Kayat Bittencourt, Ananya Panda, Yun Jiang, Junichi Tokuda, Ravi Seethamraju, Clare Tempany-Afdhal, Verena Obmann, Katherine Wright, Mark Griswold, Nicole Seiberlich, Vikas Gulani

**Affiliations:** 1 Department of Biomedical Engineering, Case Western Reserve University, Cleveland, Ohio; 2 Siemens Medical Solutions Inc, Boston, Massachusetts; 3 Department of Radiology, University Hospital and Case Western Reserve University, Cleveland, Ohio; 4 DASA company, Rio de Janeiro, RJ Brazil; 5 Department of Radiology, Mayo Clinic, Rochester, Minnesota; 6 Department of Radiology, University of Michigan, Ann Arbor, Michigan; 7 Department of Radiology, Harvard Medical School, Harvard University, Boston, Massachusetts; 8 Department of Radiology, Brigham and Women’s Hospital, Boston, Massachusetts; 9 Department of Diagnostic, Interventional and Pediatric Radiology, Inselspital Bern, University of Bern, Berne, Switzerland

**Keywords:** MR fingerprinting, prostate, quantitative mapping, repeatability, reproducibility

## Abstract

**Purpose:**

To evaluate multicenter repeatability and reproducibility of T_1_ and T_2_ maps generated using MR fingerprinting (MRF) in the International Society for Magnetic Resonance in Medicine/National Institute of Standards and Technology MRI system phantom and in prostatic tissues.

**Methods:**

MRF experiments were performed on 5 different 3 Tesla MRI scanners at 3 different institutions: University Hospitals Cleveland Medical Center (Cleveland, OH), Brigham and Women’s Hospital (Boston, MA) in the United States, and Diagnosticos da America (Rio de Janeiro, RJ) in Brazil. Raw MRF data were reconstructed using a Gadgetron-based MRF online reconstruction pipeline to yield quantitative T_1_ and T_2_ maps. The repeatability of T_1_ and T_2_ values over 6 measurements in the International Society for Magnetic Resonance in Medicine/National Institute of Standards and Technology MRI system phantom was assessed to demonstrate intrascanner variation. The reproducibility between the 4 clinical scanners was assessed to demonstrate interscanner variation. The same-day test–retest normal prostate mean T_1_ and T_2_ values from peripheral zone and transitional zone were also compared using the intraclass correlation coefficient and Bland–Altman analysis.

**Results:**

The intrascanner variation of values measured using MRF was less than 2% for T_1_ and 4.7% for T_2_ for relaxation values, within the range of 307.7 to 2360 ms for T_1_ and 19.1 to 248.5 ms for T_2_. Interscanner measurements showed that the T_1_ variation was less than 4.9%, and T_2_ variation was less than 8.1% between multicenter scanners. Both T_1_ and T_2_ values in in vivo prostatic tissue demonstrated high test–retest reliability (intraclass correlation coefficient > 0.92) and strong linear correlation (*R*^2^ > 0.840).

**Conclusion:**

Prostate MRF measurements of T_1_ and T_2_ are repeatable and reproducible between MRI scanners at different centers on different continents for the above measurement ranges.

## 1 | INTRODUCTION

MR fingerprinting (MRF)^1^ is a quantitative tissue property mapping technique that can be used to efficiently generate multiple tissue property maps simultaneously,^2–6^ and it has been applied to measure quantitative T_1_ and T_2_ measurements in the prostate.^2,7,8^ MRF has the potential to enable objective diagnosis and follow up of disease in the prostate. Previous research has shown that MRF-derived T_1_ and T_2_ values can be used to differentiate between normal peripheral zone (PZ) and prostate cancer,^2,9,10^ and in combination with apparent diffusion coefficient mapping can differentiate between low and intermediate/high grade cancers.^7,11^ MRF-based relaxometry combined with apparent diffusion coefficient mapping also improves transition zone (TZ) lesion characterization.^8,12^

In order to translate and use MRF meaningfully in clinical practice, the quantitative tissue properties measured with MRF must be repeatable and reproducible.^13^ If these features can be demonstrated, observed relaxation time differences within a tissue can be assumed to be due to differences in physiology rather than measurement variability and/or scanner instability as long as the measured differences are greater than the measurement error. MRF has been shown to provide highly reproducible quantitative maps in both 2D^14^ and 3D^15^ acquisitions. MRF-derived T_1_ and T_2_ measurements are also repeatable over time,^16^ with excellent reproducibility in vivo across different scanner types.^17,18^ Several in vivo multicenter studies demonstrated high levels of repeatability and reproducibility of MRF in the brain.^17,19^ However, repeatability and reproducibility of the prostate MRF acquisition in phantom and prostatic tissues across different centers have not yet been demonstrated.

The purpose of this study was to evaluate multicenter repeatability and reproducibility of T_1_ and T_2_ estimates based on the MRF technique using the International Society for Magnetic Resonance in Medicine/National Institute of Standards and Technology (ISMRM/NIST) MRI system phantom^20^ and prostatic tissues in patients.

## 2 | METHODS

### 2.1 | MRF data acquisition

This Health Insurance Portability and Accountability Act–compliant study was approved by the local institutional review board, and written informed consent was obtained for all in vivo scans. Experiments were performed on 5 different 3 Tesla MRI scanners (1 Skyra and 4 Verio scanners, Siemens Healthcare, Erlangen, Germany) with different software versions (VE11C, VB19, and VB17) in 3 different institutions: University Hospitals Cleveland Medical Center (UHCMC) and Brigham and Women’s Hospital (BWH) in the United States, and Diagnosticos da America (DASA) in Brazil. An MRF–fast imaging with steady-state precession acquisition designed for use in the prostate^21^ was employed with the following parameters: FOV 400 × 400 mm^2^; matrix 400 × 400; flip angles 3.38–50°;TR 11.2–14.2 ms; slice thickness 5 mm; 3000 TRs, acquisition time 39 s/slice. A delay time of 5 s was inserted between measurements to ensure sufficient magnetization recovery before beginning the next experiment for both phantom and in vivo studies.

### 2.2 | MRF dictionary simulation

In order to efficiently match each measured signal time-course to the appropriate combination of tissue property values, a precalculated MRF dictionary, which can be used as a look-up table, was generated using Bloch equation simulations in MatLab (MathWorks 2015b, Natick, MA). In the prostate region, the T_1_ is expected to range between 1000 and 2500 ms, and the T_2_ between 20 and 300 ms for 3 Tesla systems.^8,22,23^ Dictionary resolutions of T_1_ values of [10:5:90, 100:10:1000, 1020:20:1500, 1550:50:2050, 2150:100:2950] and T_2_ values of [2:2:10, 15:5:150, 160:10:200, 250:50:500], denoted by min:step:max (ms), were used to balance between matching accuracy and MRF dictionary size. The dictionary had a total of 5970 entries.

### 2.3 | MRF map reconstruction

All map reconstruction was performed using a Gadgetron MRF implementation,^24^ which was exported from UHCMC to BWH and DASA for online reconstruction at each of the institutions. The computers used to perform the reconstructions had an 8GB Nvidia GeForce GTX 1080 graphics card; a 10 core, 2.2GHz Intel Xeon E5–2630 v4 processor; and 64GB of 2400 MHz DDR4 RAM. The raw data was passed to the Gadgetron MRF reconstruction pipeline and processed using principal component analysis-based coil compression to reduce the number of coils from 8–12 to 8, as suggested in Ref. 25. To further reduce the computational load and memory requirements without reducing the performance, singular value decomposition basis compression^26^ was applied to the MRF data to compress the number of time points from 3000 to 43, which preserved 99.9% of collected information. The GPU-enabled non-uniform fast Fourier transform^27^ was then used to grid the data. Multi-coil images were combined with adaptive coil combination.^28^ Finally, cross-correlation pattern matching was applied to the data using the precalculated dictionary to extract quantitative T_1_ and T_2_ values for each voxel. The Gadgetron reconstruction took 17.8 s for each slice.

### 2.4 | Phantom study

The accuracy of the T_1_ and T_2_ values measured using MRF was validated using the T_2_ layer of ISMRM/NIST MRI system phantom with T_1_ values between 307.7 and 2360 ms and T_2_ values between 19.1 and 248.5 ms. The phantom was placed in the magnet for at least 20 min before the acquisition to reduce any errors due to motion of the water making up the phantom. Six single-slice MRF measurements were then collected, with a delay of 5 s between measurements, on all 5 scanners. Following this acquisition, data for the same-day test–retest study were collected on the UHCMC Verio 1, UHCMC Verio 2, UHCMC Skyra, and DASA Verio. The phantom was moved out of the magnet and placed again in the magnet, again allowed to settle for at least 20 min, and another set of 6 single-slice MRF acquisitions was collected. Neither B_0_ nor B_1_ maps were collected in this study. The results from the MRF measurements were compared to the reference values measured and reported by NIST.^16^

### 2.5 | In vivo prostate study

In addition to the phantom study, in vivo experiments were performed in 24 patients with suspected prostate cancer (7 patients on the UHCMC Verio 1, mean age 68.4 years, age range 67–71 years; 6 patients on the BWH Verio, mean age 67.3 years, age range 59–76 years; and 11 patients on the DASA Verio, mean age 60.7 years, age range 37–71 years). The protocol used was the same as that described for the phantom study, with the following exceptions: No settling time was required for the in vivo prostate measurements. Also, instead of single-slice measurements, 2 sets of 2-slice MRF measurements with no slice gaps were acquired to assess same-day test–retest reliability. The patients were removed from the scanner and then repositioned between the 2 MRF acquisitions.

### 2.6 | Statistical analysis

For the ISMRM/NIST MRI system phantom study, the mean and SD for each sphere were calculated from a circular region of interest (70 pixels in size with a radius of 4.7 mm) that was manually drawn on the maps. For repeatability, intrascanner variation of T_1_ and T_2_ values was assessed using the coefficient of variation (CV), defined as the ratio of the SD to the mean of 6 measurements and expressed as a percentage:

$${\text{CV}}_{\text{intrascanner}}=100\times \frac{\text{SD}\;\text{of}\;6\;\text{measurements}}{\text{mean}\;\text{of}\;6\;\text{measurements}}.$$

The intrascanner variation was calculated for each MRI scanner. For reproducibility, the coefficients of variation for T_1_ and T_2_ values between the 4 clinical scanners were calculated to demonstrate interscanner variation:

$$\begin{array}{l} {\text{CV}}_{\text{interscanner}}=100\\ \times \frac{\text{SD}\;\text{of}\;\text{measurements}\;\text{from}\;4\;\text{scanners}}{\text{mean}\;\text{of}\;\text{measurements}\;\text{from}\;4\;\text{scanners}}. \end{array}$$

The mean of all 6 measurements was first calculated for each scanner. The mean and SD across the 4 scanners were then calculated and compared to the mean and SD for measurement no. 5 to show the differences between interscanner variation from multiple measurements and a single measurement.

For the in vivo subjects, regions of interest in the PZ and TZ were drawn by a radiologist (l.k.b., with 13 years of radiology experience) in maps from both scans for all patients. Note that the ROIs from patients (10 pixels in size) were drawn in normal appearing regions (PI-RADS 1 or PI-RADS 2) with no specific findings. Mean T_1_ and T_2_ values were calculated for each region of interest. The intraclass correlation coefficient^1,3^ and Bland–Altman analysis were used to evaluate the test–retest reliability in the in vivo prostate study.

## 3 | RESULTS

**FIGURE 1:**
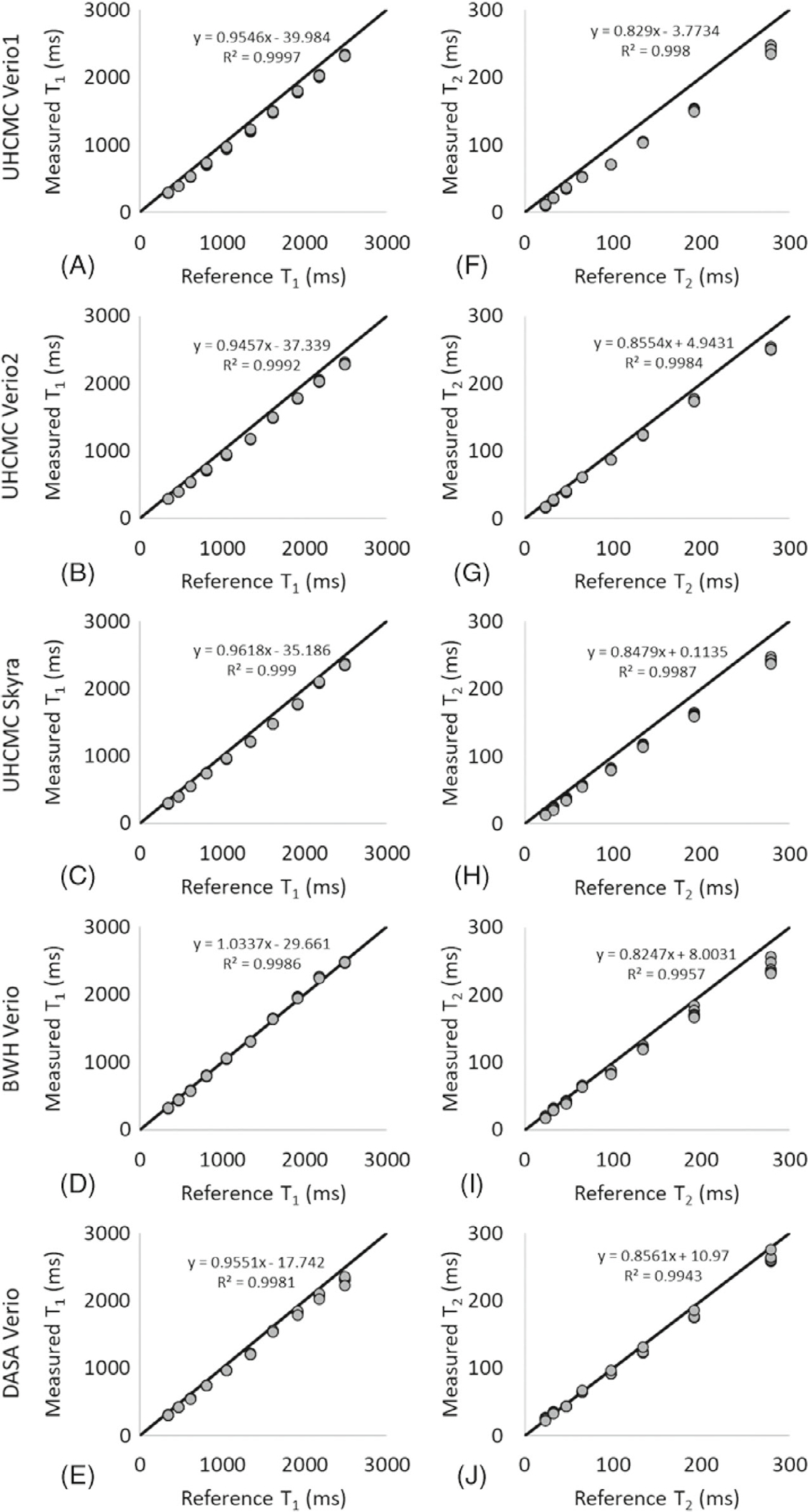
The means of the MRF-FISP T_1_ (A) and T_2_ (B) values measured on the UHCMC Verio 1, UHCMC Verio 2, UHCMC Skyra, DASA Verio, and BWH Verio using the ISMRM/NIST MRI system phantom. The MRF-FISP T_1_ and T_2_ values are compared to the reference values measured and reported by NIST. BWH, Brigham and Women’s Hospital; DASA, Diagnosticos da America; FISP, fast imaging with steady-state precession; ISMRM/NIST, International Society for Magnetic Resonance in Medicine/National Institute of Standards and Technology; MRF, MR fingerprinting; UHCMC, University Hospitals Cleveland Medical Center

The means of the 6 measurements obtained from the ISMRM/NIST MRI system phantom on 5 scanners at the 3 different medical institutions are presented in Figure 1. The *x*-axis labels are the reference values for each of the spheres as measured and reported by NIST. The results show a strong linear correlation (*R*^2^ > 0.998 for T_1_, *R*^2^ > 0.994 for T_2_) with the reference values. The bias for each vial (calculated as the difference between the measured T_1_ and T_2_ values and the reference values, divided by the reference values) for each of the 5 scanners, is shown in Supporting Figure S1.

**FIGURE 2:**
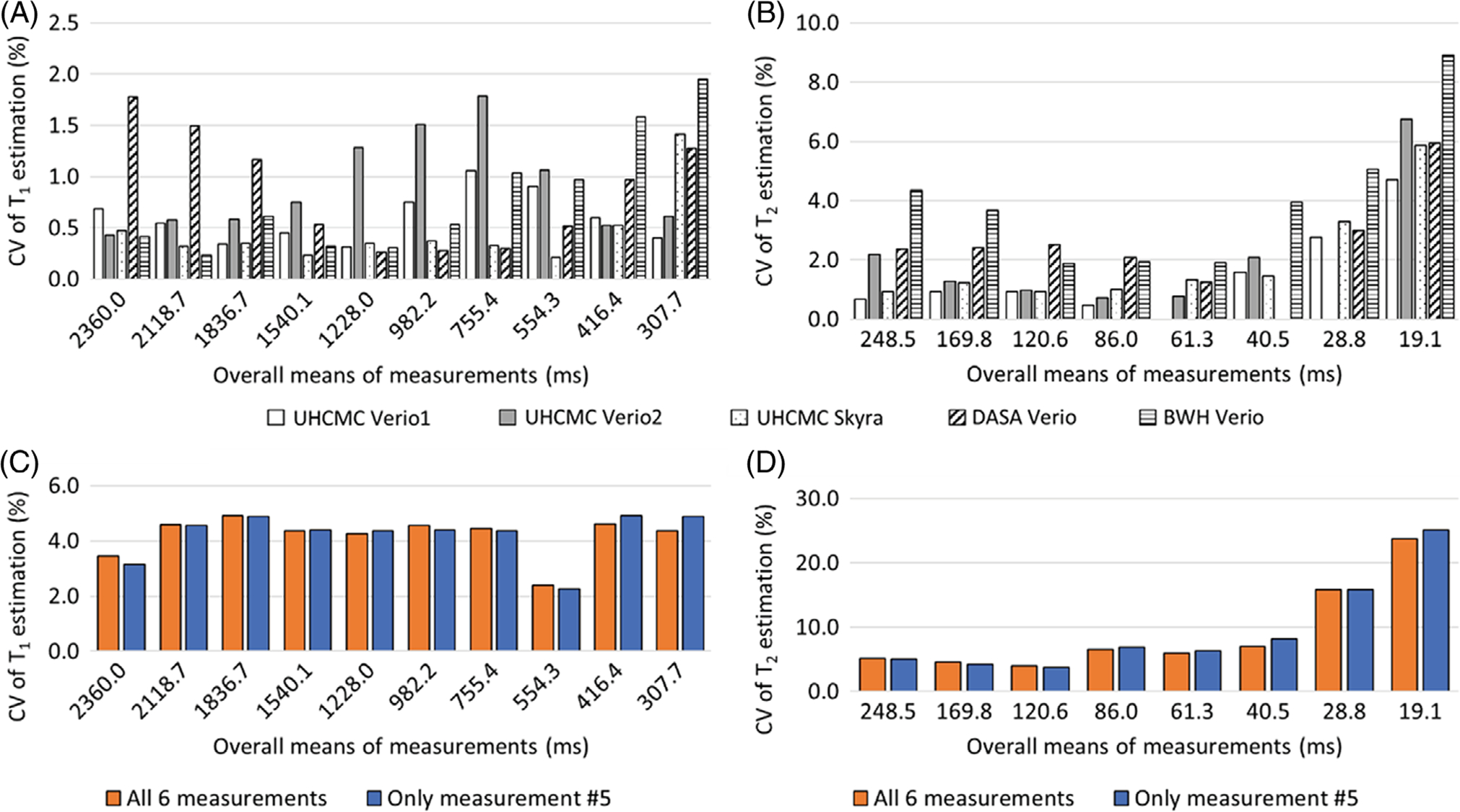
The CVs for MRF-FISP T_1_ (A) and T_2_ (B) values showing intrascanner variation on the UHCMC Verio 1 (white), UHCMC Verio 2 (gray), UHCMC Skyra (dotted), DASA Verio (oblique line), and BWH Verio (horizontal line) using the ISMRM/NIST MRI system phantom (calculated for 6 repeated measurements). The CVs for MRF-FISP T_1_ (C) and T_2_ (D) values showing interscanner variation of 4 clinical scanners over all 6 measurements (orange) and a single measurement (no. 5 of the 6 measurements, shown in blue)CV, coefficients of variation

Figure 2 shows the CV for each of the spheres with T_1_ values between 307.7 and 2360.0 ms and T_2_ values between 19.1 and 248.5 ms, as calculated by dividing the SD of the 6 repeat measurements by the mean of the 6 measurements (expressed as a percentage). Figure 2A, 2B show the intrascanner CVs for T_1_ and T_2_. The T_1_ estimates had a variation of 0.2% to 2.0%, and T_2_ estimates had a variation of 0.0% to 4.7%, with the exception of the vial with a T_2_ value of 19.1 ms, which showed a variation of 8.9%. The interscanner CVs over all 6 measurements are shown in orange in Figure 2C, 2D, and the CVs for a single measurement (no. 5 of the 6 measurements) are shown in blue. These interscanner measurements exhibited a T_1_ variation of 2.3% to 4.9% for T_1_ values between 307.7 and 2360.0 ms and T_2_ variation of 2.3% to 8.1% for T_2_ values between 40.5 and 248.5 ms. The variation increased in spheres with T_2_ values of lower than 28.8 ms. The difference between interscanner variations of multiple measurements and the variation in a single measurement is less than 2%.

**FIGURE 3:**
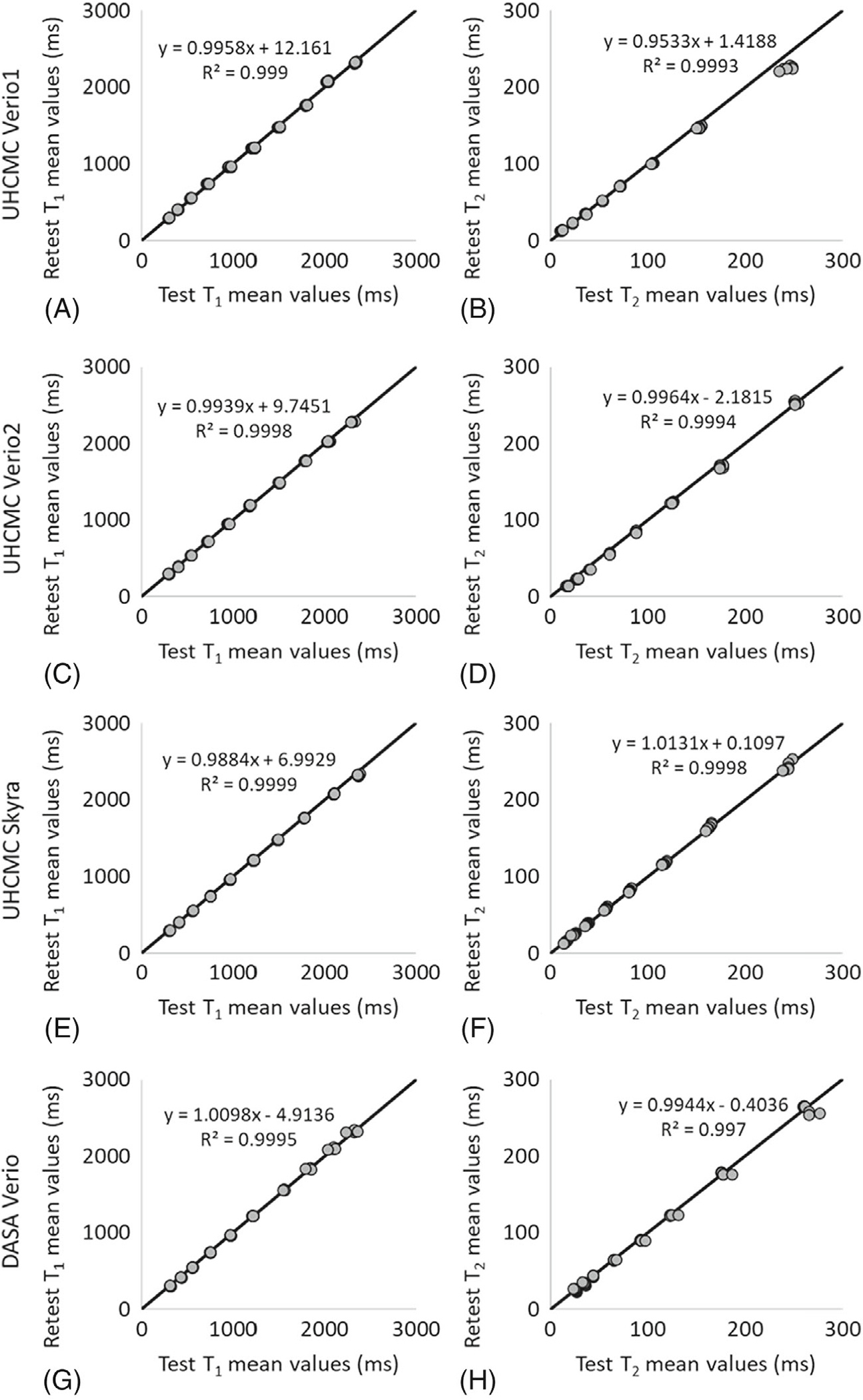
The mean T_1_ and T_2_ values for same-day test–retest reliability using the ISMRM/NIST MRI system phantom on UHCMC Verio 1 (A, B), UHCMC Verio 2 (C, D), UHCMC Skyra (E, F), and DASA Verio (G, H)

The linear correlations for both T_1_ and T_2_ values in the ISMRM/NIST MRI system phantom were above 0.99 between repeated measurements made on the UHCMC Verio 1, UHCMC Verio 2, UHCMC Skyra, and DASA Verio (Figure 3) (Supporting Figure S2).

**FIGURE 4:**
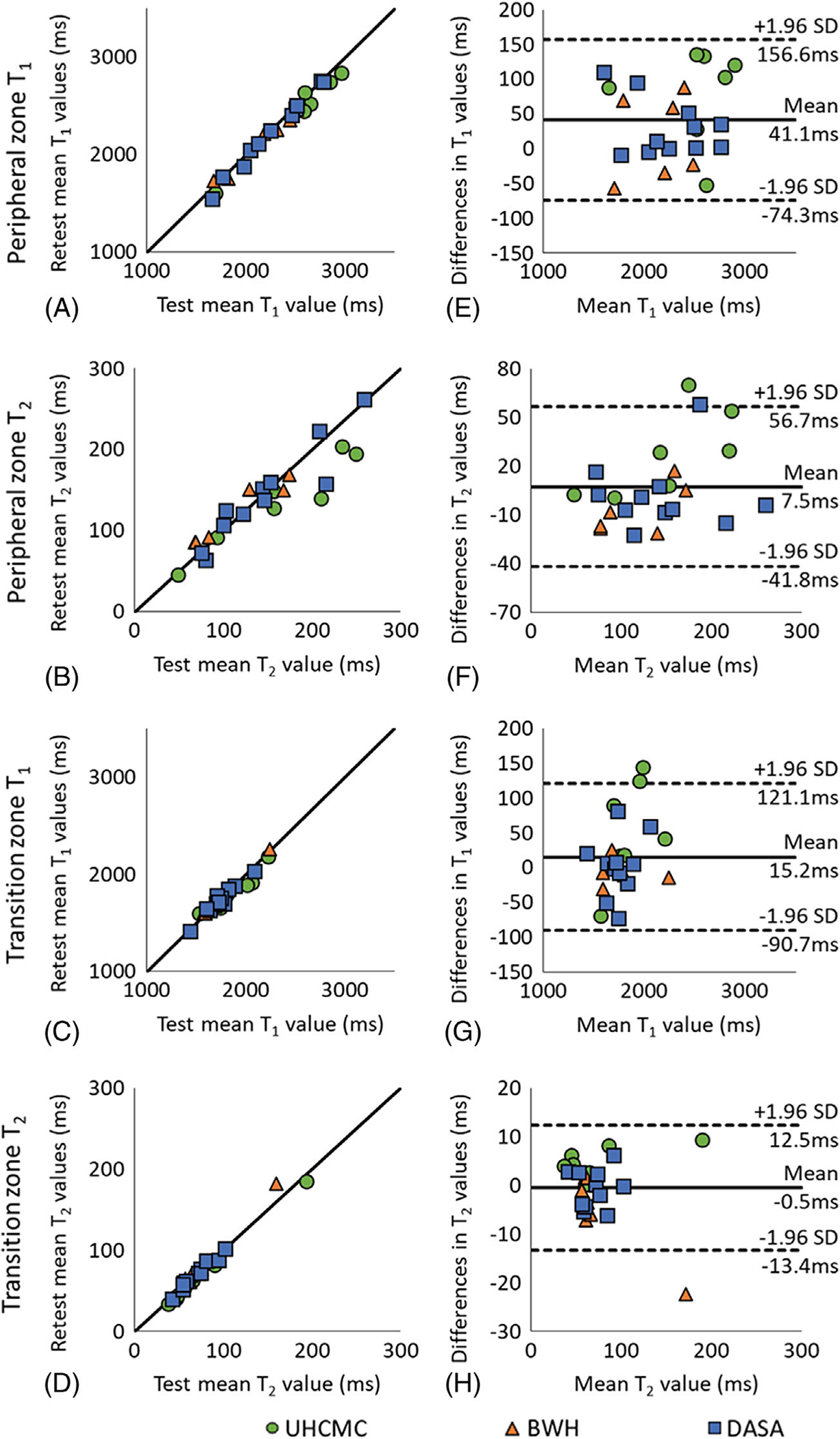
The mean T_1_ and T_2_ values for same-day test–retest reliability of patients with suspected but not confirmed prostate cancer at UHCMC Verio 1, BWH Verio, and DASA Verio measured in the peripheral zone (A, B) and transitional zone (C, D). Bland–Altman plots comparing same-day test–retest measurements of in vivo prostate peripheral zone T_1_ (E) and T_2_ (F) values and transition zone T_1_ (G) and T_2_ (H) values on UHCMC Verio 1 (green circle), BWH Verio (orange triangle), and DASA Verio (blue square)

Supporting Figure S3 shows representative prostate MRF T_1_ and T_2_ maps in patients from 5 different scanners. For the same-day test–retest in vivo prostate experiments performed on patients, the mean T_1_ and T_2_ values in both the peripheral zone and transition zones are shown in Figure 4A–4D. The mean and SD of T_1_ and T_2_ values in these zones are given in Table 1. The test–retest reliability coefficients demonstrate test–retest reliability intraclass correlation coefficient > 0.92 in both prostate regions at all 3 sites.

The Bland–Altman analysis revealed that 24 of 24 PZ T_1_ measurements, 22 of 24 PZ T_2_ and TZ T_1_ measurements, and 23 of 24 TZ T_2_ measurements fell within the 95% confidence interval (CI) for limits of agreement when difference in measurements was plotted against the mean of the measurements (Figure 4). The T_1_ values obtained from PZ and TZ demonstrated a strong linear correlation (*R*^2^ = 0.978 and *R*^2^ = 0.936, respectively) and acceptable agreement (bias 41.1 ms, 95% CI −74.3 ms to 156.6 ms; bias 15.2 ms, 95% CI −90.7 ms to 121.1 ms). The T_2_ values from PZ and TZ also showed a strong linear correlation (*R*^2^ = 0.840 and *R*^2^ = 0.970, respectively) and acceptable agreement (bias 4.5 ms, 95% CI −41.8 ms to 56.7 ms; bias −0.45 ms, 95% CI −13.4 ms to 12.5 ms) with corresponding plots presented in Figure 4F, 4H, respectively.

## 4 | DISCUSSION

This study assesses the repeatability and reproducibility of prostate MRF-derived T_1_ and T_2_ measurements on 5 different 3T MRI scanners with different software versions in 3 different medical institutions. It also demonstrates the use of a Gadgetron-based online MRF reconstruction to generate quantitative maps rapidly at the scanner. This implementation enabled the same MRF reconstruction to be used on 5 different MRI scanners in 3 different locations where the personnel had technical expertise ranging from minimal to advanced. Additionally, the improvement in the workflow made possible through the use of the online reconstruction meant that quantitative maps could be provided immediately to the radiologist for annotation and analysis. Coupled with the results demonstrating repeatability and reproducibility, this work paves the way for a Gadgetron-based MRF framework for quantitative mapping of the prostate to be distributed and used with a variety of MRI scanners around the world.

This study reports the repeatability and reproducibility of prostate MRF performed at different centers on different continents. Over the wide ranges of T_1_ and T_2_ values found in the ISMRM/NIST system phantom, intrascanner MRF T_1_ and T_2_ estimates showed small variations over 6 measurements. The interscanner measurements showed larger T_1_ and T_2_ variations between scanners at different institutions, which is similar to the results reported in Ref.^19^ These measurements are in line with other quantitative measurements in the prostate; previous research has shown that the repeatability CV for measurements of apparent diffusion coefficient in the prostate is < 2.4%, and reproducibility CV is < 4.0% across three 3T scanners.^29^ Our findings of repeatability (T_1_ CV < 2.0% and T_2_ CV < 4.7%) and reproducibility (T_1_ CV < 4.9% and T_2_ CV < 8.1%) for MRF T_1_ and T_2_ values in the phantom are similar to the reported prostate apparent diffusion coefficient values. However, T_2_ values lower than 30 ms and higher than 300 ms demonstrated larger variation. An underestimation of very high T_2_ values (> 300 ms) in the phantom study was observed as compared to reference values in Figure 1; however, variations in this range of T_2_ are not expected to be clinically relevant in the prostate because cancer and prostatitis have much shorter measured T_2_. The T_2_ step size in the MRF dictionary was set to 10 ms from 160 to 200 and 50 ms from 250 to 500 ms because such high values were not originally expected to be encountered in vivo. Finer dictionary step size and higher maximum T_2_ values in the dictionary may improve the accuracy of high T_2_ values. Similarly, the higher CV seen for vials with a T_2_ value below 30 ms likely relates to dictionary coarseness (5 ms at this range), which is a substantial fraction of the measured values. A finer dictionary with smaller step sizes could result in an improved test–retest agreement and a lower CV. Other factors that may increase systematic variation of the measured T_1_ and T_2_ values (Supporting Figure S1 and Supporting Figure S2) include temperature, B_0_ inhomogeneity, and B_1_ inhomogeneity.

In addition to the phantom experiment, this study also examined in vivo measurements in prostatic tissues. The phantom study demonstrated same-scanner test–retest reliability intraclass correlation coefficient > 0.99, whereas the in vivo study showed test–retest reliability intraclass correlation coefficient > 0.92. The slightly lower agreement in the in vivo study as compared to the phantom is likely due to a combination of patient motion, physiologic differences, dictionary coarseness, partial volume effects, and B_0_ field drift. Because the test–retest scans were performed after moving the subject, the slice selected may also be slightly different, which could add further variation to the values measured. Partial volume effects could affect the measurements, especially if evaluating small structures/lesions and smaller glands. Thinner slices with a higher spatial resolution would improve the partial volume effects in subjects with small prostates. Main magnetic field drifts could cause errors in T_2_ values. The same center frequency was used for all scans in single experiment. Adjusting center frequency before each scan may improve the reproducibility.

Differences were observed between the average T_1_ and T_2_ values of the peripheral zone in the 3 measurements from different institutions. The patient data collected from BWH showed lower T_1_ and T_2_ values as compared to the normal peripheral zone, and higher T_1_ and T_2_ values as compared to prostate cancer and noncancers reported in literature.^7^ The differences between groups likely related to differences in populations from which these cohorts were drawn. Some of the patients from BHW underwent prior biopsy or brachytherapy before MRF measurement and may have different tissue properties as compared to other 2 sites. Several patients had small or almost no PI-RADS 1 peripheral zone due to either prior therapy or benign prostatic hypertrophy (PI-RADS 2 with no specific findings); thus, peripheral zone measurements in these patients were difficult to obtain and may contain significant partial volume effects. Finally, small cohorts were scanned due to workflow pressures and distances between sites; thus, patients at each site were not from homogeneous populations. For these reasons, whereas exact matched comparisons between the patients at the 3 sites were not possible for this early study, studies with closely matched patient populations can be explored in the future.

One of the limitations in this work was the lack of age-matched healthy subjects. However, the focus of this study was on repeatability and reproducibility and not to provide normative ranges for T_1_ and T_2_ in the prostate. In order to extend the MRF results to the general population as imaging biomarkers of disease status, repeatability and reproducibility could be assessed in larger populations that include age-matched healthy subjects and patients with different pathologies.

## 5 | CONCLUSION

MRF measurements of T_1_ and T_2_ using the MRF-fast imaging with steady-state precession prostate protocol are highly repeatable and reproducible between MRI scanners at different centers on different continents.

## Supplementary Material

Lo-MRM2022- Supplementary-Material-Lo-MRM2022 **Figure S1** Percentage bias in MRF-FISP T_1_ (a) and T_2_ (b) values measured on the UHCMC Verio 1, UHCMC Verio 2, UHCMC Skyra, DASA Verio, and BWH Verio using the ISMRM/NIST MRI system phantom. The MRF-FISP T_1_ and T_2_ values are compared to the reference values measured and reported by NIST.
**Figure S2** Bland-Altman plots comparing same-day test-retest measurements using the ISMRM/NIST MRI system phantom on UHCMC Verio 1 (a and b), UHCMC Verio 2 (c and d), UHCMC Skyra (e and f), and DASA Verio (g and h).
**Figure S3** Demonstrative T_1_ and T_2_ maps generated using MRF-FISP in the prostate collected on the UHCMC Verio 1, UHCMC Verio 2, UHCMC Skyra, DASA Verio, and BWH Verio. Values in the two zones were measured from ROIs like these shown here as black circles in the PZ.

## Figures and Tables

**TABLE 1:** The mean and SD of T_1_ and T_2_ values and same-day test-retest reliability of measurements in prostatic tissue in patients in the PZ and TZ

			UHCMC Verio 1		BWH Verio		DASA Verio	
			7 Patients (68.4 ± 1.4 years)		6 Patients (67.3 ± 6.2 years)		11 Patients (60.7 ± 9.0 years)	
			Mean ± SD (ms)	ICC (95% CI)	Mean ± SD (ms)	ICC (95% CI)	Mean ± SD (ms)	ICC (95% CI)
T_1_	PZ	Test	2551.8 ± 409.7	0.99 (0.98–1.00)	2153.4 ± 331.1	0.99 (0.92–1.00)	2257.6 ± 378.9	0.98 (0.88–1.00)
		Retest	2471.9 ± 407.4		2136.6 ± 317.9		2150.6 ± 546.1	
	TZ	Test	1880.0 ± 233.3	0.96 (0.87–0.99)	1773.8 ± 244.1	0.94 (0.70–0.99)	1740.7 ± 166.0	1.00 (0.98–1.00)
		Retest	1827.6 ± 194.3		1778.8 ± 246.2		1738.2 ± 156.2	
T_2_	PZ	Test	164.1 ± 73.9	0.94 (0.78–0.98)	115.4 ± 48.7	0.92 (0.59–0.99)	146.0 ± 59.4	0.94 (0.63–0.99)
		Retest	136.1 ± 55.5		122.3 ± 38.4		143.7 ± 58.8	
	TZ	Test	77.6 ± 54.0	0.98 (0.92–0.99)	76.7 ± 40.9	1.00 (0.99–1.00)	69.3 ± 18.5	0.98 (0.89–1.00)
		Retest	72.6 ± 52.0		83.1 ± 48.8		70.0 ± 18.1	

BWH, Brigham and Women’s Hospital; CI, confidence interval; DASA, Diagnosticos da America; ICC, intraclass correlation coefficient; PZ, peripheral zone; TZ, transition zone; UHCMC, University Hospitals Cleveland Medical Center.

## Abbreviations:

MRF: MR Fingerprinting
PZ: Peripheral Zone
TZ: Transition Zone
ISMRM/NIST: International Society for Magnetic Resonance in Medicine/National Institute of Standards and Technology
UHCMC: University Hospitals Cleveland Medical Center
BWH: Brigham and Women’s Hospital
DASA: Diagnosticos da America
SVD: Singular Value Decomposition
CV: Coefficient of Variation
CI: Confidence Interval
PI-RADS: Prostate Imaging Reporting And Data System

## References

[1] Ma D, Gulani V, Seiberlich N, Magnetic resonance fingerprinting. Nature. 2013;495:187–192. 23486058 10.1038/nature11971 PMC3602925

[2] Yu AC, Badve C, Ponsky LE, Development of a combined MR fingerprinting and diffusion examination for prostate cancer. Radiology. 2017;283:729–738. 28187264 10.1148/radiol.2017161599 PMC5452885

[3] Chen Y, Jiang Y, Pahwa S, MR fingerprinting for rapid quantitative abdominal imaging. Radiology. 2016;279:278–286. 26794935 10.1148/radiol.2016152037 PMC4819902

[4] Hamilton JI, Jiang Y, Chen Y, MR fingerprinting for rapid quantification of myocardial T1, T2, and proton spin density. Magn Reson Med. 2017;77:C1. 10.1002/mrm.26216 PMC5045735 27038043

[5] Badve C, Yu A, Dastmalchian S, Magnetic resonance fingerprinting of adult brain tumors: initial experience. AJNR Am Neuroradiol. 2016;51:87–100. 10.3174/ajnr.A5035 PMC5352493 28034994

[6] European Society of Radiology (ESR). Magnetic resonance fingerprinting—a promising new approach to obtain standardized imaging biomarkers from MRI. Insights Imaging. 2015;6:163–165. 25800993 10.1007/s13244-015-0403-3 PMC4376817

[7] Panda A, O’Connor G, Lo WC, Targeted biopsy validation of peripheral zone prostate cancer characterization with magnetic resonance fingerprinting and diffusion mapping. Invest Radiol. 2019;54:485–493. 30985480 10.1097/RLI.0000000000000569 PMC6602844

[8] Panda A, Verena O, W.-C. L, MR fingerprinting and ADC mapping for characterization of lesions in transition zone of the prostate gland. Radiology. 2019;292:685–694. 31335285 10.1148/radiol.2019181705 PMC6716564

[9] Panda A, Mehta BB, Coppo S, Magnetic resonance fingerprinting-an overview. Curr Opin Biomed Eng. 2017;3:56–66. 29868647 10.1016/j.cobme.2017.11.001 PMC5984038

[10] Hsieh JJL, Svalbe I. Magnetic resonance fingerprinting: from evolution to clinical applications. J Med Radiat Sci. 2020;67:333–344. 32596957 10.1002/jmrs.413 PMC7754037

[11] Ropella-Panagis KM, Seiberlich N, Gulani V. Magnetic resonance fingerprinting: implications and opportunities for PET/MR. IEEE Trans Radiat Plasma Med Sci. 2019;3:388–399. 32864537 10.1109/trpms.2019.2897425 PMC7454032

[12] Sushentsev N, Kaggie JD, Buonincontri G, The effect of gadolinium-based contrast agent administration on magnetic resonance fingerprinting-based T(1) relaxometry in patients with prostate cancer. Sci Rep. 2020;10:20475. 33235229 10.1038/s41598-020-77331-4 PMC7686305

[13] Warntjes JB, Engström M, Tisell A, Lundberg P. Brain characterization using normalized quantitative magnetic resonance imaging. PLoS ONE. 2013;5;8:e70864. 10.1371/journal.pone.0070864 PMC3733841 23940653

[14] Ma D, Coppo S, Chen Y, Slice profile and B1corrections in 2D magnetic resonance fingerprinting. Magn Reson Med. 2017;78:1781–1789. 28074530 10.1002/mrm.26580 PMC5505861

[15] Ma D, Jiang Y, Chen Y, Fast 3D magnetic resonance fingerprinting for a whole-brain coverage. Magn Reson Med. 2018;79:2190–2197. 28833436 10.1002/mrm.26886 PMC5868964

[16] Jiang Y, Ma D, Keenan KE, Stupic KF, Gulani V, Griswold MA. Repeatability of magnetic resonance fingerprinting T1 and T2 estimates assessed using the ISMRM/NIST MRI system phantom. Magn Reson Med. 2017;78:1452–1457. 27790751 10.1002/mrm.26509 PMC5408299

[17] Körzdörfer G, Kirsch R, Liu K, Reproducibility and repeatability of MR fingerprinting Relaxometry in the human brain. Radiology. 2019;292:429–437. 31210615 10.1148/radiol.2019182360

[18] Sushentsev N, Kaggie JD, Slough RA, Carmo B, Barrett T. Reproducibility of magnetic resonance fingerprinting-based T1 mapping of the healthy prostate at 1.5 and 3.0 T: a proof-of-concept study. PLoS One. 2021;16:e0245970. 33513165 10.1371/journal.pone.0245970 PMC7846281

[19] Buonincontri G, Biagi L, Retico A, Multi-site repeatability and reproducibility of MR fingerprinting of the healthy brain at 1.5 and 3.0 T. Neuroimage. 2019;195:362–372. 30923028 10.1016/j.neuroimage.2019.03.047

[20] Stupic KF, Ainslie M, Boss MA, A standard system phantom for magnetic resonance imaging. Magn Reson Med. 2021;86:1194–1211. 33847012 10.1002/mrm.28779 PMC8252537

[21] Jiang Y, Ma D, Seiberlich N, Gulani V, Griswold MA. MR fingerprinting using fast imaging with steady state precession (FISP) with spiral readout. Magn Reson Med. 2015;74:1621–1631. 25491018 10.1002/mrm.25559 PMC4461545

[22] Simpkin CJ, Morgan VA, Giles SL, Riches SF, Parker C, de Souza NM. Relationship between T2 relaxation and apparent diffusion coefficient in malignant and non-malignant prostate regions and the effect of peripheral zone fractional volume. Br J Radiol. 2013;86:20120469. 23426849 10.1259/bjr.20120469 PMC3635787

[23] Baur ADJ, Hansen CM, Rogasch J, Evaluation of T1 relaxation time in prostate cancer and benign prostate tissue using a modified look-locker inversion recovery sequence. Sci Rep. 2020;10:3121. 32080281 10.1038/s41598-020-59942-z PMC7033189

[24] Lo W-C, Jiang Y, Franson D, Griswold M, Gulani V, Seiberlich N. MR Fingerprinting using a Gadgetron-based reconstruction. In Proceedings of the ISMRM Workshop on Magnetic Resonance Fingerprinting, Cleveland, OH, 2018.

[25] Hansen MS, Sørensen TS. Gadgetron: an open source framework for medical image reconstruction. Magn Reson Med. 2013;69:1768–1776. 22791598 10.1002/mrm.24389

[26] McGivney DF, Pierre E, Ma D, SVD compression for magnetic resonance fingerprinting in the time domain. IEEE Trans Med Imaging. 2014;33:2311–2322. 25029380 10.1109/TMI.2014.2337321 PMC4753055

[27] Sorensen TS, Schaeffter T, Noe KO, Hansen MS. Accelerating the nonequispaced fast fourier transform on commodity graphics hardware. IEEE Trans Med Imaging. 2008;27:538–547. 18390350 10.1109/TMI.2007.909834

[28] Inati SJ, Hansen MS, Kellman P. A Solution to the Phase Problem in Adaptive Coil Combination. In Proceedings of the 21st Annual Meeting of IS MRM, Salt Lake City, UT, 2013. p. 2672.

[29] Wang Y, Tadimalla S, Rai R, Quantitative MRI: defining repeatability, reproducibility and accuracy for prostate cancer imaging biomarker development. Magn Reson Imaging. 2021;77:169–179. 33388362 10.1016/j.mri.2020.12.018

