# Supplementary material for: Multicenter Repeatability and Reproducibility of MR Fingerprinting in Phantoms and in Prostatic Tissue
Source: Magn Reson Med. 2022 Jun 17;88(4):1818–27. doi: 10.1002/mrm.29264 (PMC9469467; doi:10.1002/mrm.29264)
Supplement: Supplementary file 1 — Figure S1 Percentage bias in MRF‐FISP T1 (a) and T2 (b) values measured on the UHCMC Verio 1, UHCMC Verio 2, UHCMC Skyra, DASA Verio, and BWH Verio using the ISMRM/NIST MRI system phantom. The MRF‐FISP T1 and T2 values are compared to the reference values measured and reported by NIST. Figure S2 Bland‐Altman plots comparing same‐day test‐retest measurements using the ISMRM/NIST MRI system phantom on UHCMC Verio 1 (a and b), UHCMC Verio 2 (c and d), UHCMC Skyra (e and f), and DASA Verio (g and h). Figure S3 Demonstrative T1 and T2 maps generated using MRF‐FISP in the prostate collected on the UHCMC Verio 1, UHCMC Verio 2, UHCMC Skyra, DASA Verio, and BWH Verio. Values in the two zones were measured from ROIs like these shown here as black circles in the PZ. [file MRM-88-1818-s001.docx]

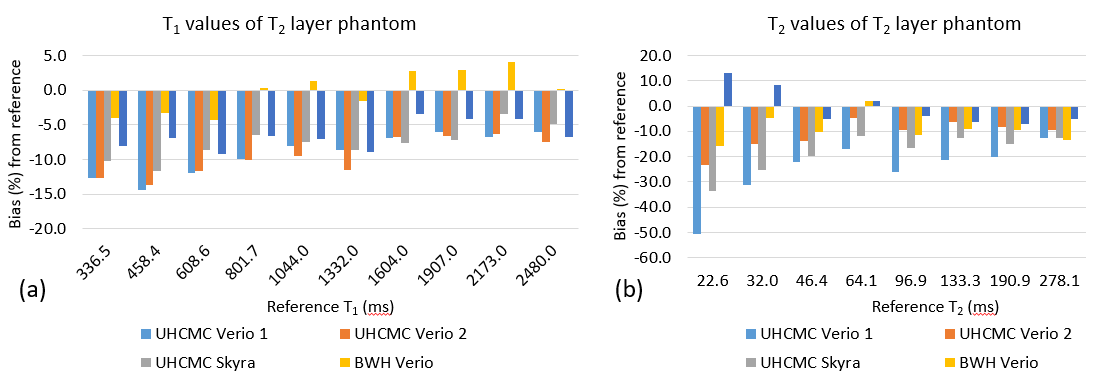


**Figure S1**. Percentage bias in MRF-FISP T_1_ (a) and T_2_ (b) values measured on the UHCMC Verio 1, UHCMC Verio 2, UHCMC Skyra, DASA Verio, and BWH Verio using the ISMRM/NIST MRI system phantom. The MRF-FISP T_1_ and T_2_ values are compared to the reference values measured and reported by NIST.


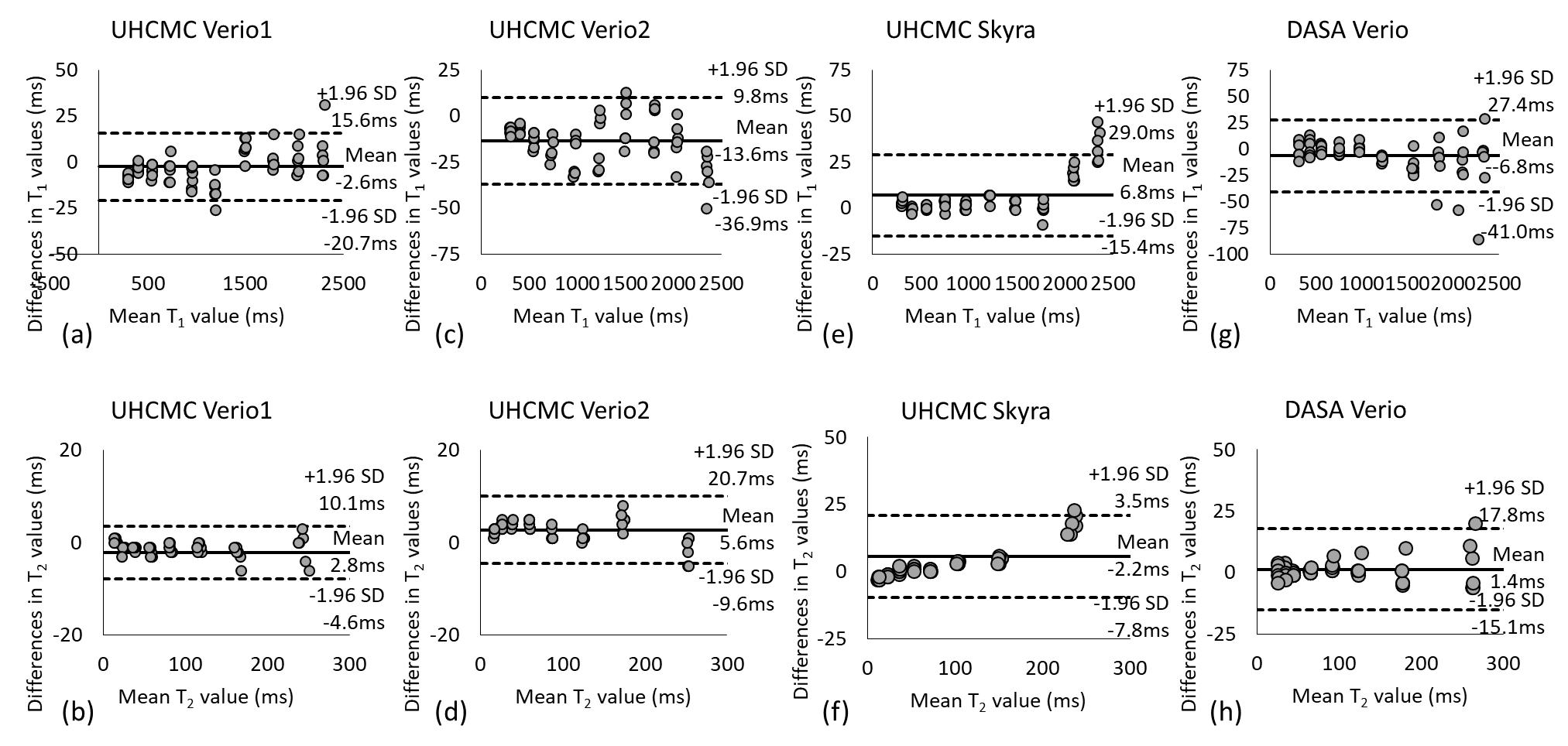


**Figure S2**. Bland-Altman plots comparing same-day test-retest measurements using the ISMRM/NIST MRI system phantom on UHCMC Verio 1 (a and b), UHCMC Verio 2 (c and d), UHCMC Skyra (e and f), and DASA Verio (g and h).


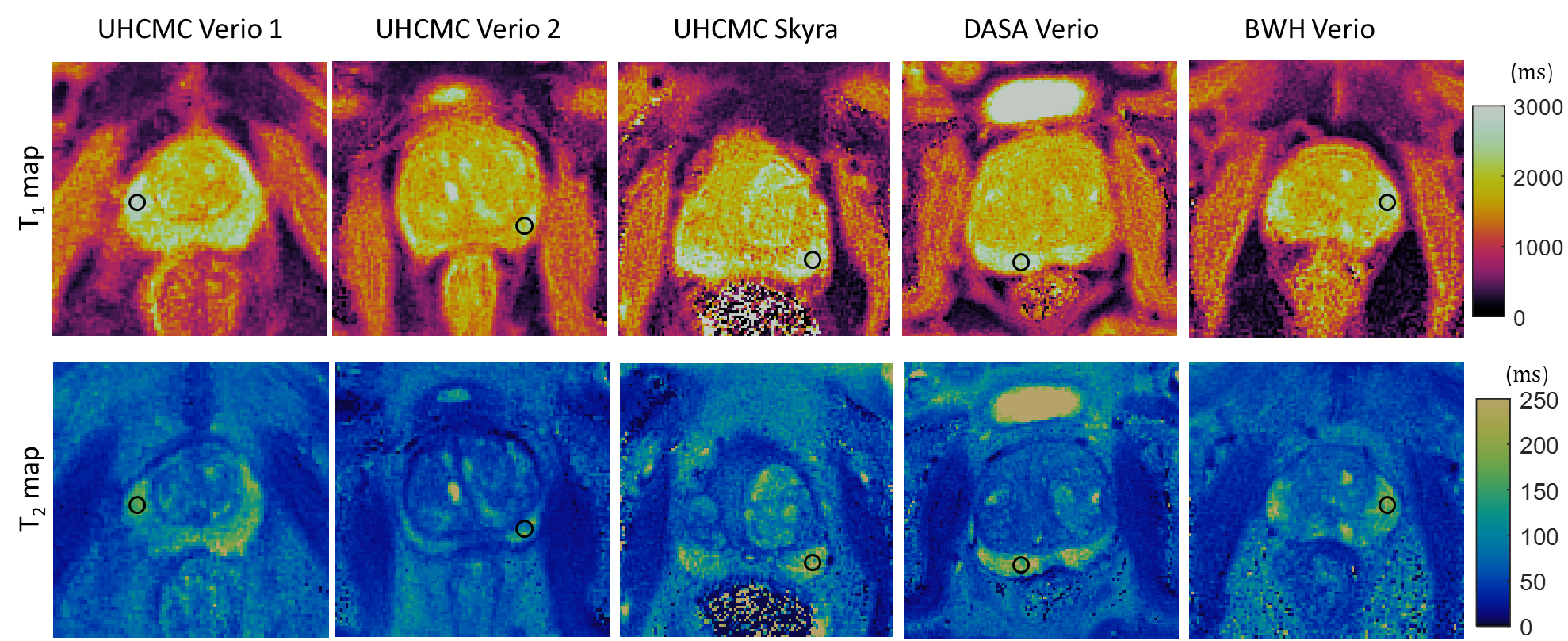


**Figure S3**. Demonstrative T_1_ and T_2_ maps generated using MRF-FISP in the prostate collected on the UHCMC Verio 1, UHCMC Verio 2, UHCMC Skyra, DASA Verio, and BWH Verio. Values in the two zones were measured from ROIs like these shown here as black circles in the PZ.
