# Supplementary material for: Multicenter Repeatability and Reproducibility of MR Fingerprinting in Phantoms and in Prostatic Tissue
Source: Magn Reson Med. Author manuscript; Available in PMC 2023 Oct 1. (PMC9469467; doi:10.1002/mrm.29264)
Supplement: Supplementary file 1 [file NIHMS1831691-supplement-Lo-MRM2022-_Supplementary-Material-Lo-MRM2022.pdf]

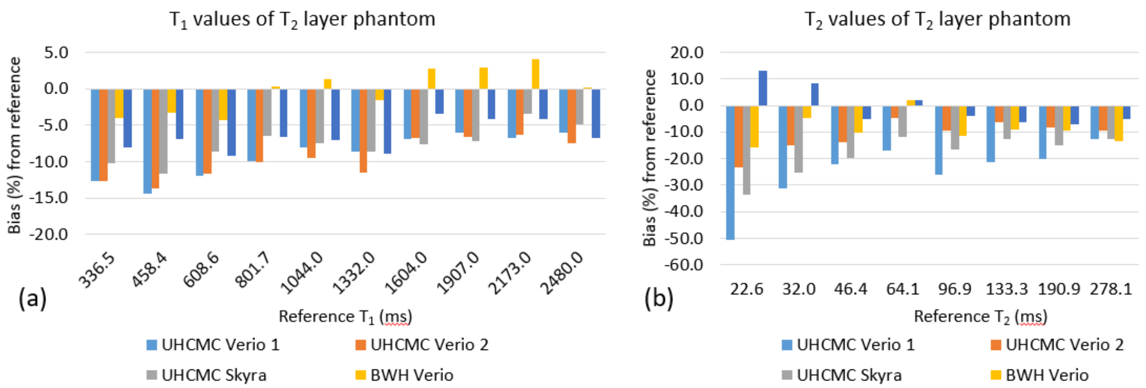

Figure S1. Percentage bias in MRF-FISP T_1 (a) and T_2 (b) values measured on the UHCMC Verio 1, UHCMC Verio 2, UHCMC Skyra, DASA Verio, and BWH Verio using the ISMRM/NIST MRI system phantom. The MRF-FISP T_1 and T_2 values are compared to the reference values measured and reported by NIST.

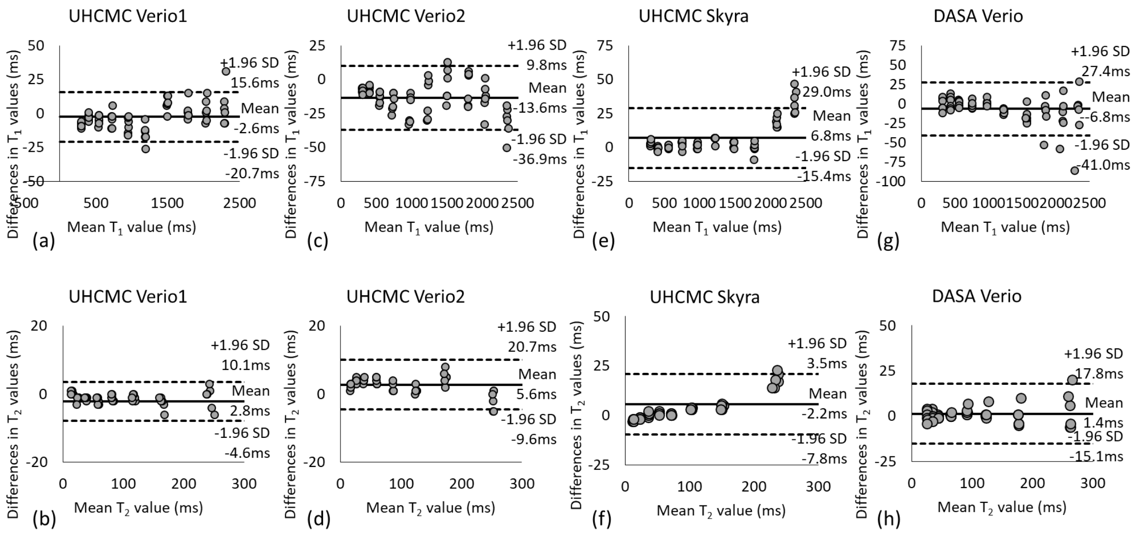

Figure S2. Bland-Altman plots comparing same-day test-retest measurements using the ISMRM/NIST MRI system phantom on UHCMC Verio 1 (a and b), UHCMC Verio 2 (c and d), UHCMC Skyra (e and f), and DASA Verio (g and h).

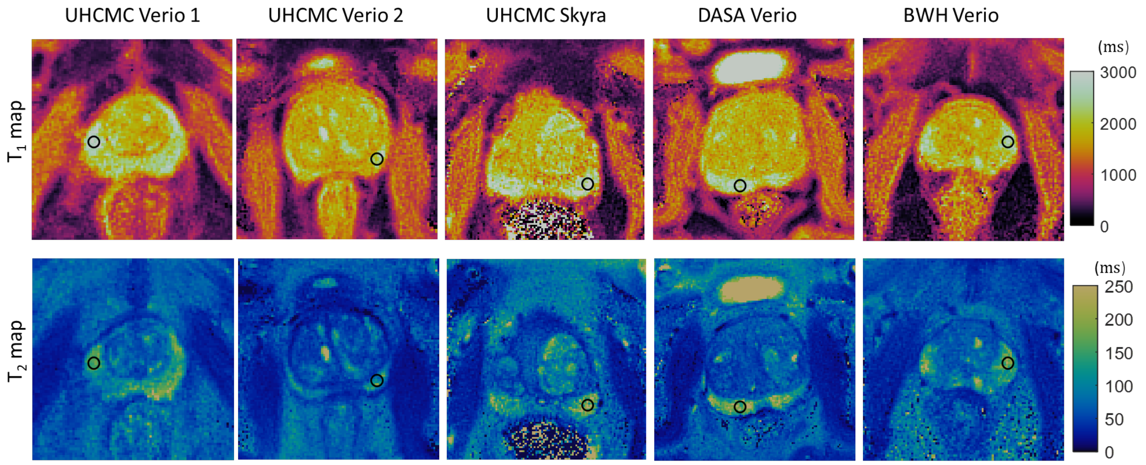

Figure S3. Demonstrative T_1 and T_2 maps generated using MRF-FISP in the prostate collected on the UHCMC Verio 1, UHCMC Verio 2, UHCMC Skyra, DASA Verio, and BWH Verio. Values in the two zones were measured from ROIs like these shown here as black circles in the PZ.
